# Circulating insulin-like growth factor-1 and brain health: Evidence from 369,711 participants in the UK Biobank

**DOI:** 10.1186/s13195-023-01288-5

**Published:** 2023-08-22

**Authors:** Zhi Cao, Jiahao Min, Qilong Tan, Keyi Si, Hongxi Yang, Chenjie Xu

**Affiliations:** 1 School of Public Health, Hangzhou Normal University, NO.2318, Yuhangtang Road, Yuhang District, Hangzhou, 311121 China; 2 School of Public Health, Zhejiang University School of Medicine, Hangzhou, China; 3 School of Public Health, Harbin Medical University, Harbin, China; 4 Department of Health Statistics, Naval Medical University, Shanghai, China; 5 School of Basic Medical Sciences, Tianjin Medical University, Tianjin, China

**Keywords:** IGF-1, Dementia, Stroke, Parkinson’s disease, Brain volume

## Abstract

**Background:**

The effects of insulin-like growth factor-1 (IGF-1) deficiency on cognitive decline have been consistently reported in animal studies, but the relationship between IGF-1 and human brain health remains controversial. Our study aimed to investigate the associations of serum IGF-1 concentrations with some brain-related disorders and neuroimaging features.

**Methods:**

This prospective study included 369,711 participants (55.8 ± 8.1 years) from the UK biobank who had serum IGF-1 measured and were free from brain-related disorders of interest — dementia, stroke, and Parkinson’s disease (PD) — at enrollment (2006–2010). Restricted cubic splines and Cox proportional hazards models were used to detect the associations between IGF-1 concentrations and brain-related diseases. In addition, general linear regressions were applied to explore the relationship between IGF-1 concentrations and neuroimaging features (volumes of white matter, grey matter, and hippocampus and white matter hyperintensity) among a sub-sample of 36,458 participants with magnetic resonance imaging data collected since 2014.

**Results:**

During a median follow-up of 12.6 years, a total of 4,857 dementia, 6,240 stroke, and 2,116 PD cases were documented. The dose–response analyses yielded U-shaped relationships between IGF-1 concentrations and risks of dementia and stroke (*P* < 0.001 for non-linearity), with the lowest risks at 18 nmol/L and 26 nmol/L, respectively. A positive linear relationship was observed between IGF-1 concentrations and risk of PD (*P* = 0.163 for non-linearity). Moreover, neuroimaging analyses showed that higher IGF-1 concentrations were associated with greater volumes of white matter (β = 2.98 × 10^–4^, *P* < 0.001) and hippocampus (β = 3.37 × 10^–4^, *P* = 0.002) and smaller white matter hyperintensity (β = -3.12 × 10^–3^, *P* < 0.001).

**Conclusions:**

Apart from the diverse associations with neuroimaging features, both low and high IGF-1 concentrations are associated with increased risks of dementia and stroke and higher IGF-1 concentrations are linked to a higher risk of PD, highlighting the potential of IGF-1 as a biomarker for risk stratification of brain health.

**Supplementary Information:**

The online version contains supplementary material available at 10.1186/s13195-023-01288-5.

## Introduction

Insulin-like growth factor-1 (IGF-1) is a peptide hormone that plays a critical role in brain health [1]. IGF-1 in the central nervous system is either produced locally in a paracrine manner or derived primarily from the liver, which stimulates cell growth and proliferation [2]. It can exert pleiotropic effects, such as regulating cerebral blood flow and promoting neurogenesis and neuroplasticity [3, 4], by binding to the IGF-1 receptors that are present throughout the brain [1]. In natural condition, plasma IGF-1 reaches a peak (approximately 52 nmol/L) around puberty and then declined progressively to approximately 13 nmol/L by the age of 75 years, in parallel with the age-related cognitive decline [5].

Previous animal studies have consistently reported favorable effects of IGF-1 on brain-related outcomes. Mice with both insulin receptor and IGF-1 receptor deleted in the hippocampus displayed impaired spatial learning and memory, mimicking the dementia phenotype [6]. An overexpression of IGF-1 induced by transgenic techniques or stimulation of sensory neurons was associated with a significantly larger brain volume and improved spatial learning, respectively [7, 8]. Besides, IGF-1 has been demonstrated to inhibit the aggregation of amyloid-β protein [9] and the phosphorylation of tau [10], two hallmarks of Alzheimer’s disease (AD). Moreover, a growing body of studies have found that AD patients have lower IGF-1 concentrations than cognitively normal individuals [11–13]. However, evidence about the relationship between IGF-1 and brain health from clinical and epidemiological studies was mixed. For instance, in the Framingham cohort (*n* = 3,582; aged 65 ± 11 years), participants with IGF-1 in the lowest quartile had a 51% greater risk of developing AD and higher IGF-1 concentrations were associated with greater brain volumes in those without dementia [14]. In contrast, results of another cohort (*n* = 286; aged 40–80 years) demonstrated that participants with baseline serum IGF-1 in the highest quintile had worse processing capacity and global cognition after 8.3 years of follow-up [15]. While in the Health In Men Study (*n* = 3,432; aged 70 + years), no association was observed between plasma concentrations of IGF-1 and incident dementia over a mean follow-up of 9.2 years [16]. There is also inconsistency in the association of IGF-1 with the risk of stroke and Parkinson's disease (PD). For stroke, some studies have shown a protective role for IGF-1 [17, 18], while others have shown no association with incident stroke [19]. For PD, most of the studies to date have focused on differences in IGF-1 levels in PD patients and healthy controls [20], but there is still a paucity of evidence on the association between IGF-1 and the risk of PD. Most of these studies were conducted among older adults with limited sample sizes (*n* < 5000). Moreover, while previous studies have assessed non-linearity by examining percentiles of IGF-1, the possibility of dose–response association of IGF-1 with the risk of dementia, stroke, and PD has not been fully characterized in a large general population sample.

To address the foregoing research gaps, the current study aimed to prospectively investigate the associations of serum IGF-1 concentrations with the risk of some brain-related disorders (dementia, stroke, and PD) using more than 0.3 million middle-aged and older participants in the UK Biobank cohort. In addition, a subgroup of participants (over 30,000) with information on magnetic resonance imaging (MRI) was used to evaluate the associations between IGF-1 concentrations and brain macrostructure (volumes of white matter, grey matter, and hippocampus and white matter hyperintensity).

## Methods

### Study design and population

This is a prospective, population-based cohort study of participants derived from the UK Biobank. Between April 2006 and December 2010, the UK Biobank recruited 502,528 adults (37–73 years old) from the general population in the UK. Participants attended one of the 22 assessment centers across England, Scotland, and Wales, where they completed nurse-led electronic questionnaires, physical examination, and biological samples collections, and agreed to be followed up of health-related outcomes through linkage to national electronic health-related datasets [21]. Furthermore, the UK Biobank aimed to re-invite 100,000 participants to undergo brain, cardiac, and abdominal magnetic resonance imaging (MRI). Data collection began in 2014 and is still ongoing. By the time of the data analyses, information for over 40,000 participants had been released [22].

In this study, we restricted the primary analysis to participants who were free of dementia, cardiovascular disease (including stroke), cancer, and PD and who had complete data on serum IGF-1 concentrations at recruitment (*N* = 369,711). From the original UK Biobank cohort (*N* = 502,528), neuroimaging analyses were further performed in a subset of 36,458 participants who were free of dementia, stroke, and PD prior to MRI assessment and had quality-controlled MRI data (Supplemental Figure S1).

### Measurement of IGF‑1

Serum IGF-1 concentrations were measured by chemiluminescent immunoassays (DiaSorin Liaison XL, analytical range 1.3–195 nmol/L) using fasting arterial blood samples drawn at recruitment and stored at − 80 °C [23]. The average within-laboratory coefficients of variation (ratio of the standard deviation [SD] to the mean) were 6.03% for low concentrations, 5.29% for medium concentrations, and 6.18% for high concentrations. Moreover, the assay of serum IGF-1 was registered with an external quality assurance (EQA) scheme (RIQAS Immunoassay Speciality 1) to verify accuracy. The EQA results showed that 100% of participated distributions (*n* = 105) were good or acceptable.

### Ascertainment of brain-related disorders

The primary outcomes for this study were the all-cause dementia, stroke and PD. Every resident in England, Scotland, and Wales has a unique National Health Service identification number, which used to link all participants to electronic health records. The diagnosis for incident dementia (F00-F03, G30-G31), stroke (I60, I61, I63, I64) and PD (G20) were coded according to the International Classification of Diseases Tenth Revision (ICD-10). For participants who were free of brain-related disorders at baseline, follow-up time was calculated as the period from the baseline to the first occurrence of dementia, stroke, or PD, the date of death, or the end of the follow-up (September 30, 2021), whichever came first.

### Neuroimaging features

A 3-Tesla, 32-channel coil Siemens Skyra scanner (Siemens Medical Solutions, Germany) was used by the UK Biobank to obtain MRI, with 1 × 1 × 1 resolution and a view field of 208 × 256 × 256. The MRI protocols have been described in detail elsewhere [22]. Briefly, numerical volume was calculated by preprocessed three-dimensional magnetization for rapid echo-gradient (3D MP-RAGE) T1-weighted image derived phenotypes. The T2-FLAIR structural imaging was undertaken in 6 min with TR 5000.0 ms, TE 395.0 ms and spatial resolution of 1.05 × 1 × 1 mm. White matter, grey matter and hippocampus volume were generated from processed T1 images, white matter hyperintensities were quantified combined analyses of T1 and T2-FLAIR data. White matter volume and grey matter volume were noted in mm^3^ and normalized for head size. Full details on structural image segmentation and data normalization are provided elsewhere [24].

### Assessment of covariates

A wide range of sociodemographic factors, lifestyle factors, familial factors and chronic diseases at recruitment were considered as potential confounders. Participants completed a detailed questionnaire on a touch-screen computer about their sociodemographic factors (age, sex, educational attainment, and ethnicity) and lifestyle factors (smoking status, frequency of alcohol intake, and consumption of vegetable and fruit). Townsend deprivation score was used as a measure of socioeconomic status and was assigned to participants based on their residential postcode at recruitment [25]. Body mass index (BMI) was calculated as weight (in kilograms) divided by height (in meters) squared. Hypertension was defined as systolic blood pressure ≥ 140 mmHg or diastolic blood pressure ≥ 90 mmHg or regular use of antihypertensive medication. Diabetes was determined if the participant had been told by a doctor about their diagnosis of diabetes. Total cholesterol and C-reactive protein in serum were analyzed using the Beckman Coulter AU5800 Platform and the Abbott Architect platform, respectively. UK Biobank genotyping was conducted by Affymetrix using the bespoke BiLEVE Axiom array and the UK Biobank Axiom array, which determines the carrier status of APOE ε4 allele, a well-established risk factor for the development of AD. Description of sample processing workflow and preparation for genotyping can be found on (https://biobank.ndph.ox.ac.uk/showcase/showcase/docs/genotyping_sample_workflow.pdf).

### Statistical analysis

The baseline characteristics of participants across IGF-1 quartiles were summarized using descriptive statistics. The mean and SD of continuous variables, and number along with proportion of categorical variables were calculated.

Cox proportional-hazards models with age as timescale were used to estimate hazard ratios (HRs) and 95% confidence intervals (CIs) for the associations between sex-age-specific quartile of IGF-1 concentrations and the risk of brain-related disorders. The proportional hazard assumption was checked by tests based on Schoenfeld residuals, and the results indicated that the assumption were not violated. To assess the different potential confounding effects on the associations of interest, we developed three models by sequential inclusion of different covariates: model 1 was adjusted for age (timescale) and sex; model 2 was further adjusted for ethnicity, educational attainment, and Townsend deprivation index; and model 3 was additionally adjusted for smoking status, alcohol intake frequency, vegetable and fruit intake, BMI, hypertension, diabetes, total cholesterol, and C-reactive protein. We used missing indicators for missing values of categorical variables and imputed those of continuous variables with mean values, as the missing percentage was very low (less than 1%).

We also used restricted cubic splines with five knots at the 5^th^, 35^th^, 50^th^, 65^th^, and 95^th^ centiles to explore the dose–response association between IGF-1 as a continuous variable and the risk of brain-related disorders. Wald Chi-square test was performed to test linearity. We would further identify the cut-off value of IGF-1 if the association was nonlinear.

Furthermore, we used general linear models to estimate the associations between IGF-1 concentrations (coded as a continuous variable and as quartiles) and neuroimaging features (volumes of white matter, grey matter, and hippocampus and white matter hyperintensity). The white matter and grey matter volumes were normalized to each participant’s head size. The potential confounders that were adjusted for were as same as those in the brain-related disorder analyses.

We performed a series of analyses to assess the robustness of our findings. First, analyses were either additionally adjusted for different cognitive performance at baseline (reaction time, fluid intelligence score, and prospective memory results), considering that cognitive decline may be a prodromal phase of dementia [26]. Second, the abovementioned process was replicated for the carrier status of APOE ε4 allele, an important impact factor for both IGF-1 concentrations and the risk of AD [27]. Third, to minimize the potential contribution of reverse causality to these findings, we did a lag analysis by excluding those with brain-related disorder onset during the first two or four years of follow-up. Fourth, multiple imputation was applied to the missing values of covariates for five times and the estimates were pooled using Rubin’s rules.

All analyses were performed using STATA 16 statistical software (StataCorp) and R i386 3.4.3 (R Foundation for Statistical Computing). All *P* values were two‐sided, and *P* < 0.05 was considered statistically significant.

## Results

### Baseline characteristics

Of the 369,711 participants included in this study, the mean (SD) age was 55.8 (8.1) years and the proportion of female was 54.9%. Table 1 shows the characteristics of the participants by sex-age-specific IGF-1 quantile. The IGF-1 quartiles were Q1: < 17.7 nmol/L, Q2: 17.7–21.4 nmol/L, Q3: 21.4–24.9 nmol/L, and Q4: > 24.9 nmol/L. During a median follow-up of 12.6 years, a total of 4,857 (1.3%), 6,240 (1.7%) and 2,116 (0.6%) individuals developed dementia, stroke and PD, respectively. In comparison with the baseline characteristics of those who were excluded from the current study, no obvious differences were observed (Supplemental Tables S1, S2).

**Table 1:** Baseline characteristics of study population by IGF-1 quartile

Characteristics	Total (*N* = 369,711)	IGF-1 quartile			
		**Q1 (*N* = 92,560)**	**Q2 (*N* = 92,482)**	**Q3 (*N* = 92,305)**	**Q4 (*N* = 92,364)**
Age (years)	55.8 (8.1)	56.2 (8.0)	55.9 (8.1)	55.7 (8.1)	55.4 (8.2)
Sex					
Male	166,779 (45.1%)	41,753 (45.1%)	41,705 (45.1%)	41,651 (45.1%)	41,670 (45.1%)
Female	202,932 (54.9%)	50,807 (54.9%)	50,777 (54.9%)	50,654 (54.9%)	50,694 (54.9%)
Ethnicity					
White	347,429 (94.0%)	86,048 (93.0%)	87,074 (94.2%)	87,122 (94.4%)	87,185 (94.4%)
Non-White	20,542 (5.6%)	5,999 (6.5%)	4,986 (5.4%)	4,761 (5.2%)	4,796 (5.2%)
Missing	1,740 (0.5%)	513 (0.6%)	422 (0.5%)	422 (0.5%)	383 (0.4%)
Educational attainment					
College or university degree	123,653 (33.4%)	27,587 (29.8%)	30,832 (33.3%)	323,71 (35.1%)	32,863 (35.6%)
Professional qualifications	184,291 (49.8%)	46,735 (50.5%)	46,242 (50.0%)	45,476 (49.3%)	45,838 (49.6%)
Others	57,544 (15.6%)	16,981 (18.3%)	14,407 (15.6%)	13,457 (14.6%)	12,699 (13.7%)
Missing	4,223 (1.1%)	1,257 (1.4%)	1,001 (1.1%)	1,001 (1.1%)	964 (1.0%)
Townsend deprivation index	-1.35 (3.06)	-1.02 (3.20)	-1.34 (3.06)	-1.49 (2.98)	-1.57 (2.95)
Smoking status					
Never	207,190 (56.0%)	48,716 (52.6%)	51,201 (55.4%)	52,820 (57.2%)	54,453 (59.0%)
Former	122,361 (33.1%)	32,073 (34.7%)	30,990 (33.5%)	30,087 (32.6%)	29,211 (31.6%)
Current	38,392 (10.4%)	11,226 (12.1%)	9,840 (10.6%)	8,990 (9.7%)	8,336 (9.0%)
Missing	1,768 (0.5%)	545 (0.6%)	451 (0.5%)	408 (0.4%)	364 (0.4%)
Alcohol frequency					
Daily or almost	75,188 (20.3%)	20,944 (22.6%)	19,755 (21.4%)	18,364 (19.9%)	16,125 (17.5%)
3–4 times/week	86,867 (23.5%)	19,811 (21.4%)	21,978 (23.8%)	22,781 (24.7%)	22,297 (24.1%)
1–2 times/week	96,128 (26.0%)	21,602 (23.3%)	23,824 (25.8%)	24,590 (26.6%)	26,112 (28.3%)
1–3 times/month	41,355 (11.2%)	9,915 (10.7%)	10,098 (10.9%)	10,292 (11.2%)	11,050 (12.0%)
Occasionally	41,300 (11.2%)	11,308 (12.2%)	9,961 (10.8%)	9,729 (10.5%)	10,302 (11.2%)
Never	28,052 (7.6%)	8,703 (9.4%)	6,657 (7.2%)	6,362 (6.9%)	6,330 (6.9%)
Missing	821 (0.2%)	277 (0.3%)	209 (0.2%)	187 (0.2%)	148 (0.2%)
Body mass index (kg/m^2^)	27.3 (4.7)	28.2 (5.5)	27.3 (4.7)	26.9 (4.3)	26.7 (4.0)
Vegetable and fruit intake (serving/day)	4.68 (3.04)	4.51 (3.06)	4.65 (3.01)	4.73 (3.01)	4.84 (3.08)
Hypertension	256,146 (69.3%)	65,902 (71.2%)	63,641 (68.8%)	63,146 (68.4%)	63,457 (68.7%)
Diabetes	16,089 (4.4%)	5,856 (6.3%)	3,556 (3.8%)	3,194 (3.5%)	3,483 (3.8%)
Total cholesterol (mmol/L)	5.76 (1.11)	5.73 (1.13)	5.79 (1.10)	5.79 (1.10)	5.74 (1.10)
C-reactive protein (mg/L)	2.51 (4.18)	3.42 (5.02)	2.49 (3.89)	2.17 (3.70)	1.95 (3.79)

### IGF-1 concentrations and risk of brain-related disorders

**Fig. 1:**
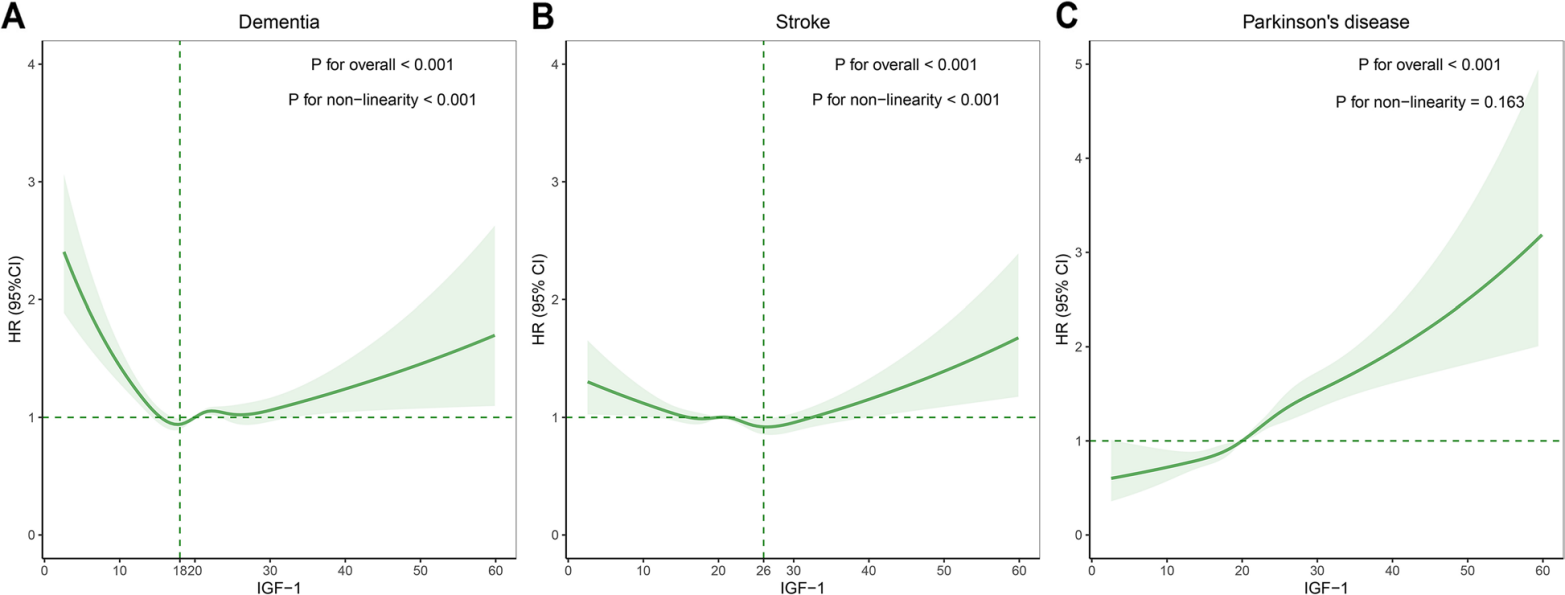
Nonlinear associations of IGF-1 concentrations with brain-related disorders. The HR for brain-related disorders with the corresponding 95% CI as a function of IGF-1 from Cox proportional hazards models adjusted for sex, age (timescale), ethnicity, educational attainment, Townsend deprivation index, smoking status, alcohol intake frequency, vegetable and fruit intake, body mass index, hypertension, diabetes, total cholesterol, and C-reactive protein. Abbreviation: CI, confidence interval; HR, hazard ratio; IGF-1, insulin-like growth factor-1

We found a U-shaped relationship between IGF-1 concentrations and the risk of dementia, with the lowest risk at a nadir IGF-1 of 18 nmol/L (*P* for non-linearity < 0.001; Fig. 1). The HR of dementia per 1-SD increment of IGF-1 (5.65 nmol/L) was 0.69 (95% CI: 0.62–0.77) in the group below 18 nmol/L, and 1.06 (95% CI: 1.01–1.11) in the group above 18 nmol/L in model 3 (Table 2). Moreover, the risk of stroke was lowest at the level of 26 nmol/L of IGF-1 and then started to increase rapidly (*P* for non-linearity < 0.001). Below 26 nmol/L, the HR of stroke per 1-SD increment of IGF-1 was 0.93 (95% CI: 0.90–0.97). Above 26 nmol/L, the HR of stroke per 1-SD increment of IGF-1 was 1.09 (95% CI: 1.02–1.17) in model 3. In contrast, we found a positive linear association between IGF-1 concentrations and the risk of PD (*P* for non-linearity = 0.163), with the HR per 1-SD increment of IGF-1 being 1.23 (95% CI: 1.18–1.28) in model 3.

**Table 2:** The associations of IGF-1 concentrations with brain-related disorders

IGF-1	Cases	Incidence rate per 1000 person-years	HR (95% CI)		
			Model 1	Model 2	Model 3
**Dementia**					
Q1	1,419	1.24 (1.18–1.31)	1.20 (1.11–1.30)	1.17 (1.08–1.26)	1.13 (1.05–1.22)
Q2	1,144	1.00 (0.94–1.05)	1.00 (Reference)	1.00 (Reference)	1.00 (Reference)
Q3	1,171	1.02 (0.96–1.08)	1.06 (0.97–1.15)	1.07 (0.99–1.16)	1.07 (0.99–1.16)
Q4	1,123	0.98 (0.92–1.04)	1.07 (0.98–1.16)	1.09 (1.01–1.18)	1.08 (1.00–1.17)
Per 1-SD increment					
IGF-1 < 18 nmol/L	2,067	1.70 (1.63–1.78)	0.61 (0.55–0.67)	0.64 (0.58–0.71)	0.69 (0.62–0.77)
IGF-1 ≥ 18 nmol/L	4,173	1.24 (1.21–1.28)	1.26 (1.17–1.35)	1.07 (1.02–1.12)	1.06 (1.01–1.11)
**Stroke**					
Q1	1,757	1.54 (1.47–1.62)	1.07 (1.00–1.15)	1.04 (0.98–1.12)	0.99 (0.93–1.06)
Q2	1,618	1.41 (1.35–1.48)	1.00 (Reference)	1.00 (Reference)	1.00 (Reference)
Q3	1,453	1.27 (1.21–1.34)	0.91 (0.85–0.98)	0.92 (0.86–0.99)	0.93 (0.87–1.00)
Q4	1,412	1.24 (1.17–1.30)	0.91 (0.85–0.98)	0.92 (0.86–0.99)	0.94 (0.87–1.01)
Per 1-SD increment					
IGF-1 < 26 nmol/L	5,300	1.44 (1.40–1.48)	0.87 (0.83–0.90)	0.89 (0.86–0.92)	0.93 (0.90–0.97)
IGF-1 ≥ 26 nmol/L	940	1.06 (0.99–1.13)	1.11 (1.03–1.18)	1.10 (1.03–1.18)	1.09 (1.02–1.17)
**Parkinson's disease**					
Q1	416	0.36 (0.33–0.40)	1.00 (Reference)	1.00 (Reference)	1.00 (Reference)
Q2	478	0.41 (0.38–0.45)	1.17 (1.03–1.33)	1.18 (1.03–1.34)	1.20 (1.05–1.37)
Q3	548	0.48 (0.44–0.52)	1.37 (1.21–1.56)	1.38 (1.21–1.57)	1.41 (1.24–1.60)
Q4	674	0.59 (0.55–0.63)	1.74 (1.54–1.97)	1.76 (1.56–1.99)	1.78 (1.57–2.01)
Per 1-SD increment	2,116	0.46 (0.44–0.48)	1.23 (1.18–1.27)	1.23 (1.18–1.28)	1.23 (1.18–1.28)

Model 1 was adjusted for age (timescale) and sex

Model 2 was additionally adjusted for ethnicity, educational attainment, Townsend deprivation index

Model 3 was additionally adjusted for smoking status, alcohol intake frequency, vegetable and fruit intake, body mass index, hypertension, diabetes, total cholesterol, and C-reactive protein

The SD of IGF-1 was 5.65 nmol/L, the cut-off value of IGF-1 quartiles were 17.7 nmol/L, 21.4 nmol/L, 24.9 nmol/L

*Abbreviation*: *CI* Confidence interval, *IGF-1* Insulin-like growth factor-1, *HR* Hazard ratio, *SD* Standard deviation

Considering the U-shaped associations of IGF-1 concentrations with dementia and stroke, the second quartile of IGF-1 was treated as the reference group when evaluating the associations between quartiles of IGF-1 and these outcomes (Table 2). Similarly, the first quartile of IGF-1 was treated as the reference group in the analysis of PD due to the linear relationship. After multivariate adjustment, IGF-1 concentrations were associated with an increased risk of dementia, with the HR being 1.13 (95% CI: 1.05–1.22) for the lowest vs. the second quartile of IGF-1 and 1.08 (95% CI: 1.00–1.17) for the highest vs. the second quartile of IGF-1 in the fully adjusted model 3. The association between IGF-1 concentrations and stroke were non-significant after adjusting for all potential confounders. Moreover, the HRs of PD for the highest vs the lowest quartile of IGF-1 was 1.74 (95% CI: 1.54–1.97), the association remained strong even when different confounders were further adjusted for (HR: 1.78; 95% CI: 1.57–2.01).

### IGF-1 concentrations and neuroimaging features

**Fig. 2:**
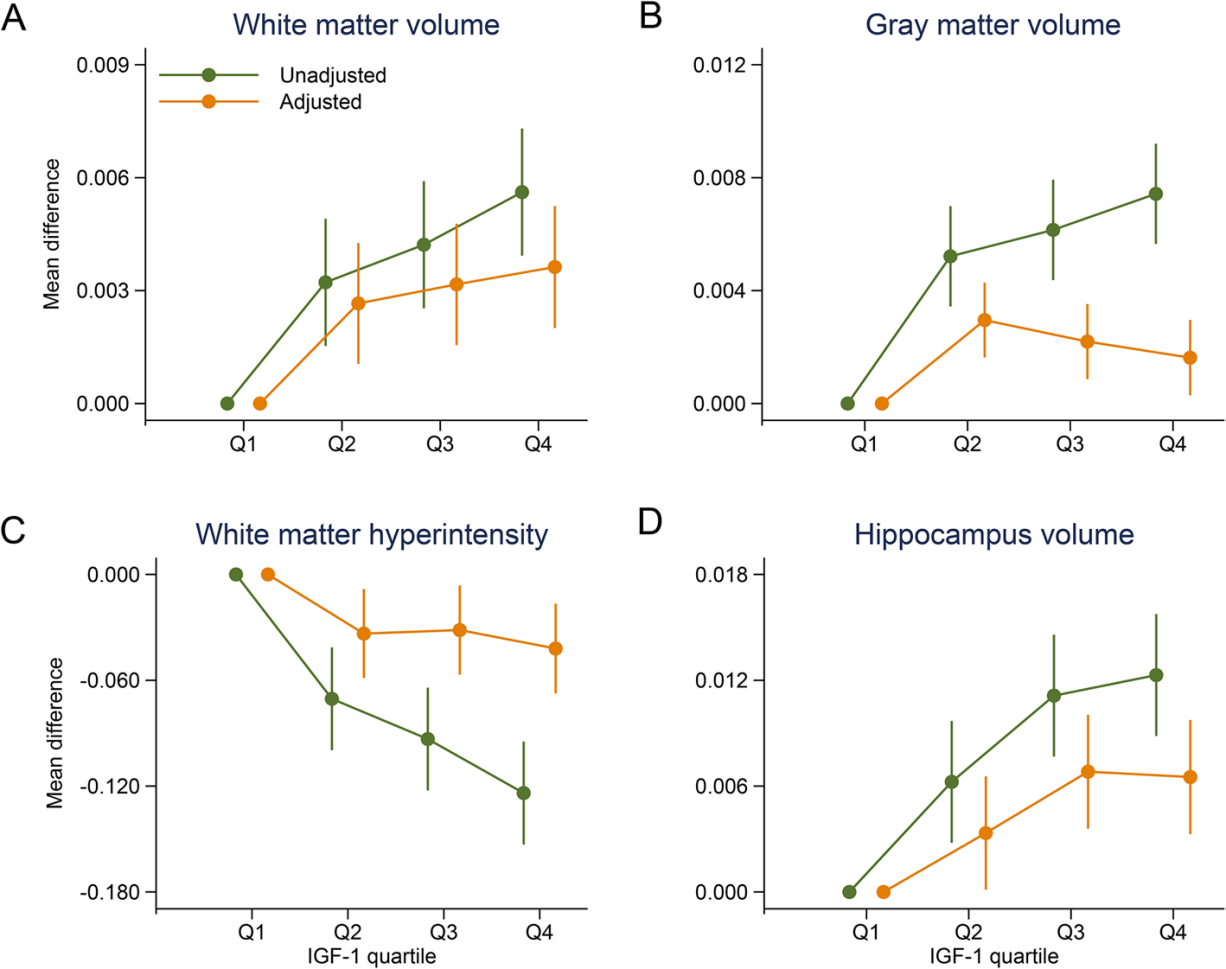
Mean differences (unadjusted and adjusted) in neuroimaging features by different quartiles of IGF-1 concentrations compared to the lowest group. Adjusted estimates were controlled for sex, age (timescale), ethnicity, educational attainment, Townsend deprivation index, smoking status, alcohol intake frequency, vegetable and fruit intake, body mass index, hypertension, diabetes, total cholesterol, and C-reactive protein

After multivariate adjustment, higher IGF-1 concentrations were associated with greater white matter volume (β = 2.98 × 10^–4^, *P* < 0.001) and hippocampal volume (β = 3.37 × 10^–4^, *P* = 0.002). Conversely, we found that higher IGF-1 concentration was associated with a smaller white matter hyperintensity (β = -3.12 × 10^–3^, *P* < 0.001), but non-significant association was observed with the grey matter volume (β = -7.36 × 10^–5^, *P* = 0.106) (Supplemental Figure S2, Supplemental Table S3). Compared to the lowest IGF-1 quartile, the adjusted mean difference in the highest IGF-1 quartile was 0.0037 (*P* < 0.001) for white matter volume, 0.0016 (*P* = 0.017) for grey matter volume, 0.0065 (*P* < 0.001) for hippocampal volume, and -0.0420 (*P* = 0.001) for white matter hyperintensity (Fig. 2).

### Sensitivity analysis

The main results were not materially changed if the models (1) were additionally adjusted for cognitive performance (Supplemental Table S4); (2) were additionally adjusted for APOE ε4 status (Supplemental Figure S3); (3) excluded the incidence of dementia, stroke or PD occurring within two or four years after recruitment (Supplemental Table S5); 4) used a multiple imputation approach to impute the missing covariates (Supplemental Figure S4).

## Discussion

This represents the largest prospective study to date that assessed the associations of serum circulating IGF-1 concentrations with brain health. We found a U-shaped relationship between IGF-1 concentrations and the risk of dementia and stroke – both high and low IGF-1 concentrations implying a higher risk. Higher IGF-1 concentrations were positively associated with an increased risk of PD, yielding a dose–response manner. Furthermore, higher IGF-1 concentrations were associated with greater volumes of white matter and hippocampus and smaller white matter hyperintensity. The findings contribute to the growing body of evidence that serum IGF-1 concentrations are crucial for risk stratification of brain health.

### IGF-1 concentrations and brain-related disorders

Less is known about the prospective associations between IGF-1 concentrations and risk of dementia in a general population. A previous study of 3582 participants from Framingham reported that lower IGF-1 concentrations were associated with an increased risk of dementia [14]. The population from this study was older on average compared to our sample, which may partly explain the differing results. Some clinical trials found that increased IGF-1 concentrations induced by exercise led to improved cognitive functions [28, 29]. On the contrary, a cross-sectional and longitudinal study including 3967 men showed that IGF-1 was not associated with either prevalent or incident dementia [16]. The dose–response association was not formally evaluated in these studies. In our study, notably, we observed a threshold effect where both lower and higher IGF-1 concentrations were associated with an increased risk of dementia. However, the disparities between the current study and previous studies may be partly attributable to differences in age distributions, IGF-1 distributions, and modeling strategies. Further research is needed to clarify the shape of the association and potential effect modifiers.

Accumulating evidence has demonstrated the neuroprotective role of IGF-1 in animal models. Earlier onset and increased incidence of neurological deficits were observed in IGF-1-deficient mice induced by liver-specific knockdown of IGF-1 [30], while boosting brain expression of IGF-1 through adenovirus-mediated transduction has been proved to protect hippocampal neurons from the toxicity of amyloid-β oligomers and prevent memory loss in an AD mouse model [31]. In terms of the underlying mechanisms, on the one hand, IGF-1 deficiency may enhance oxidative stress in endothelial cells and astrocytes, leading to neurovascular uncoupling in AD [32]; on the other hand, IGF-1 can increase amyloid-β clearance by enhancing transport of amyloid-β-carrier proteins into the brain [9]. However, few studies have uncovered the detrimental effect of high IGF-1 concentrations on the risk of dementia. In a cohort of 286 participants aged 40–80 years, those with baseline serum IGF-1 in the highest quintile had worse processing capacity and global cognition after 8.3 years of follow-up [15]. In the UK biobank, APOE ε4, a risk factor for AD, was correlated with increased IGF-1 [27]. Therefore, more studies are required to explore the mechanism and further confirm our findings.

A recent meta-analysis consisting of 2,277 patients from 17 studies revealed that a higher serum IGF-1 concentration was significantly associated with a lower risk of ischemic stroke [33]. However, our study firstly found that a lower IGF-1 concentration was moderately associated with a higher risk of stroke and a higher IGF-1 concentration might slightly increase the risk of stroke. Our novel finding provides a basis for in-depth clinical research on the relationship between IGF-1 concentrations and stroke, which is of great significance to stroke prevention, early diagnosis, and prognosis. Besides, we have not found any prospective study that examined the association between IGF-1 concentrations and the risk of PD. Nevertheless, numerous basic and translational data have appointed direct protective and homeostasis roles of IGF-1 in all brain cells, hence, IGF-1 was hypothesized to involve in the PD development [34]. Our study confirmed a positive relationship between IGF-1 concentrations and the risk of PD, which could be plausible due to IGF-1’s involvement in nerve growth and formation [35]. If our finding can be replicated by others, serum IGF-1 concentrations may serve as an important biomarker in PD risk assessment, providing a new insight for PD prevention.

### IGF-1 concentrations and neuroimaging features

To our knowledge, this is the largest cohort study to investigate the associations between IGF-1 concentrations and neuroimaging features. We found significant relationships between higher IGF-1 concentrations and greater volumes of white matter and hippocampus and smaller white matter hyperintensity. Consistent with our study, a prior study from the Framingham Heart Study suggested that a higher IGF-1 concentration was associated with larger hippocampal volume, independent of vascular risk factors [36]. Though another two studies reported a null association [14, 37]. Meanwhile, we found that higher IGF-1 concentrations were related to lower white matter hyperintensity, suggesting a potential protective role of IGF-1 not only again neurodegeneration, but also against cerebrovascular disease. IGF-1 has been shown to modulate hippocampal neurogenesis, apoptosis, and angiogenesis, which could explain part of the protective role in preserving the hippocampus and protecting from vascular insults leading to white matter hyperintensity [36]. Moreover, lower IGF-1 concentrations have been found to be associated with the presence of traditional vascular risk factors, particularly obesity, insulin resistance, and diabetes mellitus [3, 38], which might be feasible explanation for brain atrophy. Although studies on the association between IGF-1 concentrations and neuroimaging features appear to suggest a potential role in brain morphology, further well-designed studies are required to clarify these associations and possible molecular mechanisms.

## Strengths and limitations

Our study has several strengths. First, the UK Biobank is a well-designed cohort with a relatively large sample size, long-term follow-up, and accurate assessment of brain-related disorders and neuroimaging features. Second, biochemistry assays of IGF-1 were performed in a single dedicated central laboratory by a standard, reliable method with strict quality control procedures. Third, restricted cubic spline models were used to examine nonlinear associations between IGF-1 concentrations and brain-related disorders. Despite these strengths, several limitations of the current study need to be considered. First, as is typical of prior studies, circulating IGF-1 concentrations were measured at a single time point and may not reflect a long-term exposure. Because of regression dilution, studies based on a single measurement of IGF-1 at baseline could underestimate the true association between IGF-1 concentrations and outcomes of interest [39]. Second, IGF-1–related proteins such as IGF-2 and IGF-binding proteins, which play a role in the regulation of IGF-1 bioavailability and signaling [40], were not measured in this study. Therefore, the observed associations might partially reflect other aspects of the IGF-1 signaling pathway. Third, although we adjusted for potential confounders, our results are not ideal for drawing firm conclusions about the causality and directionality of the associations between IGF-1 and brain-related disorders. Future longitudinal studies and randomized controlled trials aimed at evaluating the relationships of IGF-1 with brain-related disorders are needed to clarify these effects. Fourth, the UK Biobank is not representative of the general population because UK Biobank participants are socioeconomically and health-wise "better off" than the average Briton, which may result in a lower prevalence of neurological diseases or conditions. However, it can be used to provide valid estimates of exposure-disease relationships due to its large sample size and multitude of exposures. Estimated relative risks derived from the UK Biobank are consistent with those derived from more representative population-based cohorts [41, 42]. Fifth, as our analyses on neuroimaging features relied solely on single point time data, we could not investigate the changes in neuroimaging over time.

## Conclusion

In summary, the findings from this large population-based study suggest U-shaped associations of circulating IGF-1 concentrations with the risk of dementia and stroke and a linear association with that of PD. Our findings extend previous knowledge by identifying both high and low IGF-1 concentrations as being risk factors in the development of dementia and stroke in general population. Given the lack of effective biomarkers and treatment for these overburdened diseases, the potential predictive value of serum IGF-1 concentration is of great significance for risk assessment and prevention, which warrants further studies. Besides, more research is needed to fully understand the mechanisms and to examine whether an intervention targeting IGF-1 concentration would improve brain health.

### Supplementary Information

**Additional file 1:** **Table S1.** Characteristics of participants included in or excluded from the main analysis. **Table S2.** Characteristics of participants included in or excluded from the neuroimaging analysis. **Table S3.** The associations of IGF-1 concentrations with neuroimaging features. **Table S4.** The associations of IGF-1 concentrations with brain-related disorders after additional adjustment for cognitive performance at baseline. **Table S5.** The associations of IGF-1 concentrations with brain-related disorders after excluding the first two or four years of cases during the follow-up. **Table S6.** Disease definitions. **Figure S1.** Participants flow diagram. **Figure S2.** Associations between IGF-1 concentrations and neuroimaging features. Raw data (i.e., unadjusted) are plotted. Solid lines represent estimated regression lines and shaded areas represent 95% CIs. Statistical values were obtained using multiple linear regressions controlling for sex, age, ethnicity, educational attainment, Townsend deprivation index, smoking status, alcohol intake frequency, vegetable and fruit intake, body mass index, hypertension, diabetes, total cholesterol, and C-reactive protein. Brain volume values are normalized for head size and log-transformed. **Figure S3.** The associations of IGF-1 concentrations with brain-related disorders after additional adjustment for APOE ε4 genotype. The analyses were adjusted for sex, age (timescale), ethnicity, educational attainment, Townsend deprivation index, smoking status, alcohol intake frequency, vegetable and fruit intake, body mass index, hypertension, diabetes, total cholesterol, and C-reactive protein. **Figure S4.** The associations of IGF-1 concentrations with brain-related disorders after imputing missing covariates using multiple imputation. The analyses were adjusted for sex, age (timescale), ethnicity, educational attainment, Townsend deprivation index, smoking status, alcohol intake frequency, vegetable and fruit intake, body mass index, hypertension, diabetes, total cholesterol, and C-reactive protein.

## Data Availability

The data that support the findings of this study are available from UK Biobank project site, subject to registration and application process. Further details can be found at https://www.ukbiobank.ac.uk.

## References

[1] Dyer AH, Vahdatpour C, Sanfeliu A, Tropea D (2016). The role of Insulin-Like Growth Factor 1 (IGF-1) in brain development, maturation and neuroplasticity. Neuroscience.

[2] Pollak M (2008). Insulin and insulin-like growth factor signalling in neoplasia. Nat Rev Cancer.

[3] Sandhu MS, Heald AH, Gibson JM, Cruickshank JK, Dunger DB, Wareham NJ (2002). Circulating concentrations of insulin-like growth factor-I and development of glucose intolerance: a prospective observational study. Lancet.

[4] Sonntag WE, Lynch CD, Cooney PT, Hutchins PM (1997). Decreases in cerebral microvasculature with age are associated with the decline in growth hormone and insulin-like growth factor 1. Endocrinology.

[5] van Dam PS, Aleman A (2004). Insulin-like growth factor-I, cognition and brain aging. Eur J Pharmacol.

[6] Soto M, Cai W, Konishi M, Kahn CR (2019). Insulin signaling in the hippocampus and amygdala regulates metabolism and neurobehavior. Proc Natl Acad Sci U S A.

[7] Carson MJ, Behringer RR, Brinster RL, McMorris FA (1993). Insulin-like growth factor I increases brain growth and central nervous system myelination in transgenic mice. Neuron.

[8] Harada N, Narimatsu N, Kurihara H, Nakagata N, Okajima K (2009). Stimulation of sensory neurons improves cognitive function by promoting the hippocampal production of insulin-like growth factor-I in mice. Transl Res.

[9] Carro E, Trejo JL, Gomez-Isla T, LeRoith D, Torres-Aleman I (2002). Serum insulin-like growth factor I regulates brain amyloid-beta levels. Nat Med.

[10] Bedse G, Di Domenico F, Serviddio G, Cassano T (2015). Aberrant insulin signaling in Alzheimer's disease: current knowledge. Front Neurosci.

[11] Xu LZ, Li FY, Li BQ, Cao SM, Li Y, Xu J, Jia JP (2021). Decreased Levels of Insulin-Like Growth Factor-1 Are Associated with Alzheimer's Disease: A Meta-Analysis. J Alzheimers Dis.

[12] Rui-Hua C, Yong-de P, Xiao-Zhen J, Chen J, Bin Z (2019). Decreased Levels of Serum IGF-1 and Vitamin D Are Associated With Cognitive Impairment in Patients With Type 2 Diabetes. Am J Alzheimers Dis Other Demen.

[13] Duron E, Funalot B, Brunel N, Coste J, Quinquis L, Viollet C, Belmin J, Jouanny P, Pasquier F, Treluyer JM (2012). Insulin-like growth factor-I and insulin-like growth factor binding protein-3 in Alzheimer's disease. J Clin Endocrinol Metab.

[14] Westwood AJ, Beiser A, Decarli C, Harris TB, Chen TC, He XM, Roubenoff R, Pikula A, Au R, Braverman LE (2014). Insulin-like growth factor-1 and risk of Alzheimer dementia and brain atrophy. Neurology.

[15] Tumati S, Burger H, Martens S, van der Schouw YT, Aleman A (2016). Association between Cognition and Serum Insulin-Like Growth Factor-1 in Middle-Aged & Older Men: An 8 Year Follow-Up Study. PLoS One.

[16] Almeida OP, Hankey GJ, Yeap BB, Paul Chubb SA, Gollege J, Flicker L (2018). Risk of prevalent and incident dementia associated with insulin-like growth factor and insulin-like growth factor-binding protein 3. Mol Psychiatry.

[17] Saber H, Himali JJ, Beiser AS, Shoamanesh A, Pikula A, Roubenoff R, Romero JR, Kase CS, Vasan RS, Seshadri S (2017). Serum Insulin-Like Growth Factor 1 and the Risk of Ischemic Stroke: The Framingham Study. Stroke.

[18] Johnsen SP, Hundborg HH, Sørensen HT, Orskov H, Tjønneland A, Overvad K, Jørgensen JO (2005). Insulin-like growth factor (IGF) I, -II, and IGF binding protein-3 and risk of ischemic stroke. J Clin Endocrinol Metab.

[19] Kaplan RC, McGinn AP, Pollak MN, Kuller LH, Strickler HD, Rohan TE, Cappola AR, Xue X, Psaty BM (2007). Association of total insulin-like growth factor-I, insulin-like growth factor binding protein-1 (IGFBP-1), and IGFBP-3 levels with incident coronary events and ischemic stroke. J Clin Endocrinol Metab.

[20] Li DH, He YC, Quinn TJ, Liu J (2015). Serum Insulin-Like Growth Factor-1 in Patients with De Novo, Drug Naïve Parkinson's Disease: A Meta-Analysis. PLoS One.

[21] Sudlow C, Gallacher J, Allen N, Beral V, Burton P, Danesh J, Downey P, Elliott P, Green J, Landray M (2015). UK biobank: an open access resource for identifying the causes of a wide range of complex diseases of middle and old age. PLoS Med.

[22] Miller KL, Alfaro-Almagro F, Bangerter NK, Thomas DL, Yacoub E, Xu JQ, Bartsch AJ, Jbabdi S, Sotiropoulos SN, Andersson JLR (2016). Multimodal population brain imaging in the UK Biobank prospective epidemiological study. Nat Neurosci.

[23] Elliott P, Peakman TC (2008). The UK Biobank sample handling and storage protocol for the collection, processing and archiving of human blood and urine. Int J Epidemiol.

[24] Alfaro-Almagro F, Jenkinson M, Bangerter NK, Andersson JLR, Griffanti L, Douaud G, Sotiropoulos SN, Jbabdi S, Hernandez-Fernandez M, Vallee E (2018). Image processing and Quality Control for the first 10,000 brain imaging datasets from UK Biobank. Neuroimage.

[25] Townsend P PP, Beattie A. Health and deprivation: inequality and the North. London: Croom Helm; 1988.

[26] Weuve J, Proust-Lima C, Power MC, Gross AL, Hofer SM, Thiébaut R, Chêne G, Glymour MM, Dufouil C (2015). Guidelines for reporting methodological challenges and evaluating potential bias in dementia research. Alzheimers Dement.

[27] Ferguson AC, Tank R, Lyall LM, Ward J, Celis-Morales C, Strawbridge R, Ho F, Whelan CD, Gill J, Welsh P (2020). Alzheimer's Disease Susceptibility Gene Apolipoprotein E (APOE) and Blood Biomarkers in UK Biobank (N = 395,769). J Alzheimers Dis.

[28] Smith PJ, Mabe SM, Sherwood A, Doraiswamy PM, Welsh-Bohmer KA, Burke JR, Kraus WE, Lin PH, Browndyke JN, Babyak MA (2020). Metabolic and Neurocognitive Changes Following Lifestyle Modification: Examination of Biomarkers from the ENLIGHTEN Randomized Clinical Trial. J Alzheimers Dis.

[29] Cho SY, Roh HT. Taekwondo Enhances Cognitive Function as a Result of Increased Neurotrophic Growth Factors in Elderly Women. Int J Environ Res Public Health 2019;16(6):962. 10.3390/ijerph16060962 PMC6466246 30889827

[30] Tarantini S, Valcarcel-Ares NM, Yabluchanskiy A, Springo Z, Fulop GA, Ashpole N, Gautam T, Giles CB, Wren JD, Sonntag WE (2017). Insulin-like growth factor 1 deficiency exacerbates hypertension-induced cerebral microhemorrhages in mice, mimicking the aging phenotype. Aging Cell.

[31] Selles MC, Fortuna JTS, Zappa-Villar MF, de Faria YPR, Souza AS, Suemoto CK, Leite REP, Rodriguez RD, Grinberg LT, Reggiani PC (2020). Adenovirus-Mediated Transduction of Insulin-Like Growth Factor 1 Protects Hippocampal Neurons from the Toxicity of Aβ Oligomers and Prevents Memory Loss in an Alzheimer Mouse Model. Mol Neurobiol.

[32] Tarantini S, Tran CHT, Gordon GR, Ungvari Z, Csiszar A (2017). Impaired neurovascular coupling in aging and Alzheimer's disease: Contribution of astrocyte dysfunction and endothelial impairment to cognitive decline. Exp Gerontol.

[33] Li Y, Yang W, Li J, Zhang Y, Zhang L, Chen S, He L, Zhang Y (2022). Relationship between serum insulin-like growth factor 1 levels and ischaemic stroke: a systematic review and meta-analysis. BMJ Open.

[34] Castilla-Cortázar I, Aguirre GA, Femat-Roldán G, Martín-Estal I, Espinosa L (2020). Is insulin-like growth factor-1 involved in Parkinson's disease development?. J Transl Med.

[35] Rabinovsky ED (2004). The multifunctional role of IGF-1 in peripheral nerve regeneration. Neurol Res.

[36] Wittfeld K, Raman MR, Conner SC, Aslam A, Teumer A, Nauck M, Hosten N, Habes M, DeCarli C, Vasan RS (2022). Insulin-Like Growth Factor, Inflammation, and MRI Markers of Alzheimer's Disease in Predominantly Middle-Aged Adults. J Alzheimers Dis.

[37] Salzmann A, James SN, Williams DM, Richards M, Cadar D, Schott JM, Coath W, Sudre CH, Chaturvedi N, Garfield V (2021). Investigating the Relationship Between IGF-I, IGF-II, and IGFBP-3 Concentrations and Later-Life Cognition and Brain Volume. J Clin Endocrinol Metab.

[38] Berryman DE, Glad CA, List EO, Johannsson G (2013). The GH/IGF-1 axis in obesity: pathophysiology and therapeutic considerations. Nat Rev Endocrinol.

[39] Clarke R, Shipley M, Lewington S, Youngman L, Collins R, Marmot M, Peto R (1999). Underestimation of risk associations due to regression dilution in long-term follow-up of prospective studies. Am J Epidemiol.

[40] Firth SM, Baxter RC (2002). Cellular actions of the insulin-like growth factor binding proteins. Endocr Rev.

[41] Batty GD, Gale CR, Kivimaki M, Deary IJ, Bell S. Comparison of risk factor associations in UK Biobank against representative, general population based studies with conventional response rates: prospective cohort study and individual participant meta-analysis. BMJ 2020;368:m131. 10.1136/bmj.m131 PMC7190071 32051121

[42] Fry A, Littlejohns TJ, Sudlow C, Doherty N, Adamska L, Sprosen T, Collins R, Allen NE (2017). Comparison of Sociodemographic and Health-Related Characteristics of UK Biobank Participants With Those of the General Population. Am J Epidemiol.

