# Supplementary material for: Circulating insulin-like growth factor-1 and brain health: Evidence from 369,711 participants in the UK Biobank
Source: Alzheimers Res Ther. 2023 Aug 22;15:140. doi: 10.1186/s13195-023-01288-5 (PMC10463341; doi:10.1186/s13195-023-01288-5)
Supplement: Supplementary file 1 — Additional file 1: Table S1. Characteristics of participants included in or excluded from the main analysis. Table S2. Characteristics of participants included in or excluded from the neuroimaging analysis. Table S3. The associations of IGF-1 concentrations with neuroimaging features. Table S4. The associations of IGF-1 concentrations with brain-related disorders after additional adjustment for cognitive performance at baseline. Table S5. The associations of IGF-1 concentrations with brain-related disorders after excluding the first two or four years of cases during the follow-up. Table S6. Disease definitions. Figure S1. Participants flow diagram. Figure S2. Associations between IGF-1 concentrations and neuroimaging features. Raw data (i.e., unadjusted) are plotted. Solid lines represent estimated regression lines and shaded areas represent 95% CIs. Statistical values were obtained using multiple linear regressions controlling for sex, age, ethnicity, educational attainment, Townsend deprivation index, smoking status, alcohol intake frequency, vegetable and fruit intake, body mass index, hypertension, diabetes, total cholesterol, and C-reactive protein. Brain volume values are normalized for head size and log-transformed. Figure S3. The associations of IGF-1 concentrations with brain-related disorders after additional adjustment for APOE ε4 genotype. The analyses were adjusted for sex, age (timescale), ethnicity, educational attainment, Townsend deprivation index, smoking status, alcohol intake frequency, vegetable and fruit intake, body mass index, hypertension, diabetes, total cholesterol, and C-reactive protein. Figure S4. The associations of IGF-1 concentrations with brain-related disorders after imputing missing covariates using multiple imputation. The analyses were adjusted for sex, age (timescale), ethnicity, educational attainment, Townsend deprivation index, smoking status, alcohol intake frequency, vegetable and fruit intake, body mass index, hypertensio [file 13195_2023_1288_MOESM1_ESM.doc]

**Supplemental materials**

**Table S1: Characteristics of participants included in or excluded from the main analysis**

| **Characteristics** | **Included (N=369,711)** | **Excluded**  **(N=132,817)** |
| --- | --- | --- |
| Age (years) | 55.8 (8.1) | 58.6 (7.7) |
| Sex |  |  |
| Male | 202,932 (54.9%) | 70,465 (53.1%) |
| Female | 166,779 (45.1%) | 62,352 (46.9%) |
| Ethnicity |  |  |
| White | 347,429 (94.0%) | 125,288 (94.3%) |
| Non-White | 20,542 (5.6%) | 6,492 (4.9%) |
| Missing | 1,740 (0.5%) | 1,037 (0.8%) |
| Educational attainment |  |  |
| College or university degree | 123,653 (33.4%) | 37,518 (28.2%) |
| Professional qualifications | 184,291 (49.8%) | 61,656 (46.4%) |
| Others | 57,544 (15.6%) | 27,731 (20.9%) |
| Missing | 4,223 (1.1%) | 5912 (4.5%) |
| Townsend deprivation index | -1.4 (3.1) | -1.1 (3.2) |
| Smoking status |  |  |
| Never | 207,190 (56.0%) | 66,343 (50.0%) |
| Former | 122,361 (33.1%) | 50,705 (38.2%) |
| Current | 38,392 (10.4%) | 14,587 (11.0%) |
| Missing | 1,768 (0.5%) | 1182 (0.9%) |
| Alcohol frequency |  |  |
| Daily or almost | 75,188 (20.3%) | 26,582 (20.0%) |
| 3-4 times/week | 86,867 (23.5%) | 28,576 (21.5%) |
| 1-2 times/week | 96,128 (26.0%) | 33,168 (25.0%) |
| 1-3 times/month | 41,355 (11.2%) | 14,503 (10.9%) |
| Occasionally | 41,300 (11.2%) | 16,712 (12.6%) |
| Never | 28,052 (7.6%) | 12,595 (9.5%) |
| Missing | 821 (0.2%) | 681 (0.5%) |
| Body mass index (kg/m^2^) | 27.3 (4.7) | 27.9 (5.0) |
| Vegetable and fruit intake (serving/day) | 4.7 (3.1) | 4.8 (3.2) |
| Hypertension | 256,146 (69.3%) | 92,574 (69.7%) |
| Diabetes | 16,089 (4.4%) | 10,664 (8.0%) |
| Total cholesterol (mmol/L) | 5.8 (1.1) | 5.4 (1.2) |
| C-reactive protein (mg/L) | 2.5 (4.2) | 2.9 (5.0) |

**Table S2: Characteristics of participants included in or excluded from the neuroimaging analysis**

| **Characteristics** | **Excluded (N=465,887)** | **Included**  **(N=36,641)** |
| --- | --- | --- |
| Age (years) | 56.6 (8.1) | 55.0 (7.5) |
| Sex |  |  |
| Male | 254,033 (54.5%) | 19,364 (52.8%) |
| Female | 211,854 (45.5%) | 17,277 (47.2%) |
| Ethnicity |  |  |
| White | 437,229 (93.8%) | 35,488 (96.9%) |
| Non-White | 25,976 (5.6%) | 1,058 (2.9%) |
| Missing | 2,682 (0.6%) | 95 (0.3%) |
| Educational attainment |  |  |
| College or university degree | 144,076 (30.9%) | 17,095 (46.7%) |
| Professional qualifications | 228,863 (49.1%) | 17,084 (46.6%) |
| Others | 82,929 (17.8%) | 2,346 (6.4%) |
| Missing | 10,019 (2.2%) | 116 (0.3%) |
| Townsend deprivation index | -1.2 (3.1) | -1.9 (2.7) |
| Smoking status |  |  |
| Never | 251,244 (53.9%) | 22,289 (60.8%) |
| Former | 161,035 (34.6%) | 12,031 (32.8%) |
| Current | 50,735 (10.9%) | 2,244 (6.1%) |
| Missing | 2,873 (0.6%) | 77 (0.2%) |
| Alcohol frequency |  |  |
| Daily or almost | 93,500 (20.1%) | 8,270 (22.6%) |
| 3-4 times/week | 105,175 (22.6%) | 10,268 (28.0%) |
| 1-2 times/week | 119,900 (25.7%) | 9,396 (25.6%) |
| 1-3 times/month | 51,833 (11.1%) | 4,025 (11.0%) |
| Occasionally | 55,028 (11.8%) | 2,984 (8.1%) |
| Never | 38,961 (8.4%) | 1,686 (4.6%) |
| Missing | 1,490 (0.3%) | 12 (0.0%) |
| Body mass index (kg/m^2^) | 27.5 (4.8) | 26.5 (4.2) |
| Vegetable and fruit intake (serving/day) | 4.7 (3.1) | 4.8 (2.8) |
| Hypertension | 325,119 (69.8%) | 23,601 (64.4%) |
| Diabetes | 25,821 (5.5%) | 932 (2.5%) |
| Total cholesterol (mmol/L) | 5.7 (1.1) | 5.7 (1.1) |
| C-reactive protein (mg/L) | 2.6 (4.4) | 2.0 (3.6) |

**Table S3: The associations of IGF-1 concentrations with neuroimaging features.**

|  |  | White matter volume | |  | Grey matter volume | |  | White matter hyperintensity | |  | Hippocampal volume | |
| --- | --- | --- | --- | --- | --- | --- | --- | --- | --- | --- | --- | --- |
|  |  | β | P value |  | β | P value |  | β | P value |  | β | P value |
| IGF-1 | |  |  |  |  |  |  |  |  |  |  |  |
|  | Continuous | 2.98E-04 | <0.001 |  | 7.36E-05 | 0.106 |  | -3.12E-03 | <0.001 |  | 3.37E-04 | 0.002 |
|  | Categorized |  |  |  |  |  |  |  |  |  |  |  |
|  | Q1 | 0 (Reference) |  |  | 0 (Reference) |  |  | 0 (Reference) |  |  | 0 (Reference) |  |
|  | Q2 | 2.66E-03 | 0.001 |  | 2.96E-03 | <0.001 |  | -3.35E-02 | 0.009 |  | 3.34E-03 | 0.042 |
|  | Q3 | 3.17E-03 | <0.001 |  | 2.19E-03 | 0.001 |  | -3.15E-02 | 0.015 |  | 6.82E-03 | <0.001 |
|  | Q4 | 3.63E-03 | <0.001 |  | 1.62E-03 | 0.017 |  | -4.20E-02 | 0.001 |  | 6.51E-03 | <0.001 |

Multiple linear regressions were used and controlled for sex, age, ethnicity, educational attainment, Townsend deprivation index, smoking status, alcohol intake frequency, vegetable and fruit intake, body mass index, hypertension, diabetes, total cholesterol, and C-reactive protein. Brain volume values are normalized for head size and log-transformed.

**Table S4: The associations of IGF-1 concentrations with brain-related disorders after additional adjustment for cognitive performance at baseline.**

| IGF-1 | | Adjusted for reaction time | Adjusted for fluid intelligence score | Adjusted for prospective memory results |
| --- | --- | --- | --- | --- |
| **Dementia** | |  |  |  |
|  | Q1 | 1.09 (0.94,1.28) | 1.09 (0.93,1.27) | 1.09 (0.93,1.27) |
|  | Q2 | 1 (Reference) | 1 (Reference) | 1 (Reference) |
|  | Q3 | 1.04 (0.88,1.22) | 1.03 (0.87,1.21) | 1.04 (0.88,1.22) |
|  | Q4 | 1.09 (0.93,1.28) | 1.08 (0.92,1.27) | 1.09 (0.93,1.28) |
| **Stroke** | |  |  |  |
|  | Q1 | 1.08 (0.94,1.23) | 1.08 (0.94,1.23) | 1.08 (0.94,1.23) |
|  | Q2 | 1 (Reference) | 1 (Reference) | 1 (Reference) |
|  | Q3 | 1.09 (0.96,1.25) | 1.09 (0.95,1.25) | 1.09 (0.96,1.25) |
|  | Q4 | 1.04 (0.91,1.20) | 1.04 (0.91,1.20) | 1.04 (0.91,1.20) |
| **Parkinson's disease** | |  |  |  |
|  | Q1 | 1 (Reference) | 1 (Reference) | 1 (Reference) |
|  | Q2 | 1.23 (0.96,1.59) | 1.24 (0.96,1.59) | 1.23 (0.96,1.59) |
|  | Q3 | 1.43 (1.12,1.83) | 1.43 (1.12,1.82) | 1.43 (1.12,1.82) |
|  | Q4 | 1.78 (1.40,2.25) | 1.77 (1.40,2.25) | 1.77 (1.40,2.25) |

The analyses were adjusted for sex, age (timescale), ethnicity, educational attainment, Townsend deprivation index, smoking status, alcohol intake frequency, vegetable and fruit intake, body mass index, hypertension, diabetes, total cholesterol, C-reactive protein, cognitive performance (reaction time, fluid intelligence score, or prospective memory results).

**Table S5: The associations of IGF-1 concentrations with brain-related disorders after excluding the first two or four years of cases during the follow-up.**

| IGF-1 | | First 2 years | |  | First 4 years | |
| --- | --- | --- | --- | --- | --- | --- |
|  |  | HR (95% CI) | *P* value |  | HR (95% CI) | *P* value |
| **Dementia** | |  |  |  |  |  |
|  | Q1 | 1.13 (1.04-1.22) | 0.003 |  | 1.10 (1.02-1.20) | 0.016 |
|  | Q2 | 1 (Reference) |  |  | 1 (Reference) |  |
|  | Q3 | 1.08 (0.99-1.17) | 0.068 |  | 1.09 (1.00-1.18) | 0.046 |
|  | Q4 | 1.08 (0.99-1.17) | 0.081 |  | 1.08 (0.99-1.17) | 0.084 |
| Per 1-SD increment | |  |  |  |  |  |
|  | IGF-1 < 18 nmol/l | 0.71 (0.64-0.79) | <0.001 |  | 0.74 (0.66-0.82) | <0.001 |
|  | IGF-1 > 18 nmol/l | 1.06 (1.01-1.11) | 0.024 |  | 1.06 (1.01-1.11) | 0.021 |
| **Stroke** | |  |  |  |  |  |
|  | Q1 | 0.97 (0.91-1.05) | 0.491 |  | 0.96 (0.89-1.04) | 0.298 |
|  | Q2 | 1 (Reference) |  |  | 1 (Reference) |  |
|  | Q3 | 0.93 (0.87-1.01) | 0.076 |  | 0.94 (0.87-1.01) | 0.1 |
|  | Q4 | 0.95 (0.88-1.02) | 0.164 |  | 0.95 (0.88-1.03) | 0.213 |
| Per 1-SD increment | |  |  |  |  |  |
|  | IGF-1 < 26 nmol/l | 0.93 (0.90-0.97) | <0.001 |  | 0.94 (0.90-0.97) | <0.001 |
|  | IGF-1 > 26 nmol/l | 1.09 (1.01-1.17) | 0.022 |  | 1.09 (1.01-1.17) | 0.023 |
| **Parkinson's disease** | |  |  |  |  |  |
|  | Q1 | 1 (Reference) |  |  |  |  |
|  | Q2 | 1.18 (1.03-1.35) | 0.015 |  | 1.20 (1.04-1.38) | 0.013 |
|  | Q3 | 1.38 (1.21-1.58) | <0.001 |  | 1.37 (1.20-1.58) | <0.001 |
|  | Q4 | 1.73 (1.52-1.96) | <0.001 |  | 1.70 (1.49-1.95) | <0.001 |
| Per 1-SD increment | | 1.23 (1.18-1.27) | <0.001 |  | 1.23 (1.18-1.28) | <0.001 |

The analyses were adjusted for sex, age (timescale), ethnicity, educational attainment, Townsend deprivation index, smoking status, alcohol intake frequency, vegetable and fruit intake, body mass index, hypertension, diabetes, total cholesterol, and C-reactive protein.

**Table S6: Disease definitions**

| **Brain-related disorders** | **Data fields** | **Field names** | **Data codes** |
| --- | --- | --- | --- |
| Dementia | 41202 | Diagnoses – main ICD 10 | F00-F03, G30-G31 |
| Stroke | 41202 | Diagnoses – main ICD 10 | I60, I61, I63, I64 |
| Parkinson’s disease | 41202 | Diagnoses – main ICD 10 | G20 |

**
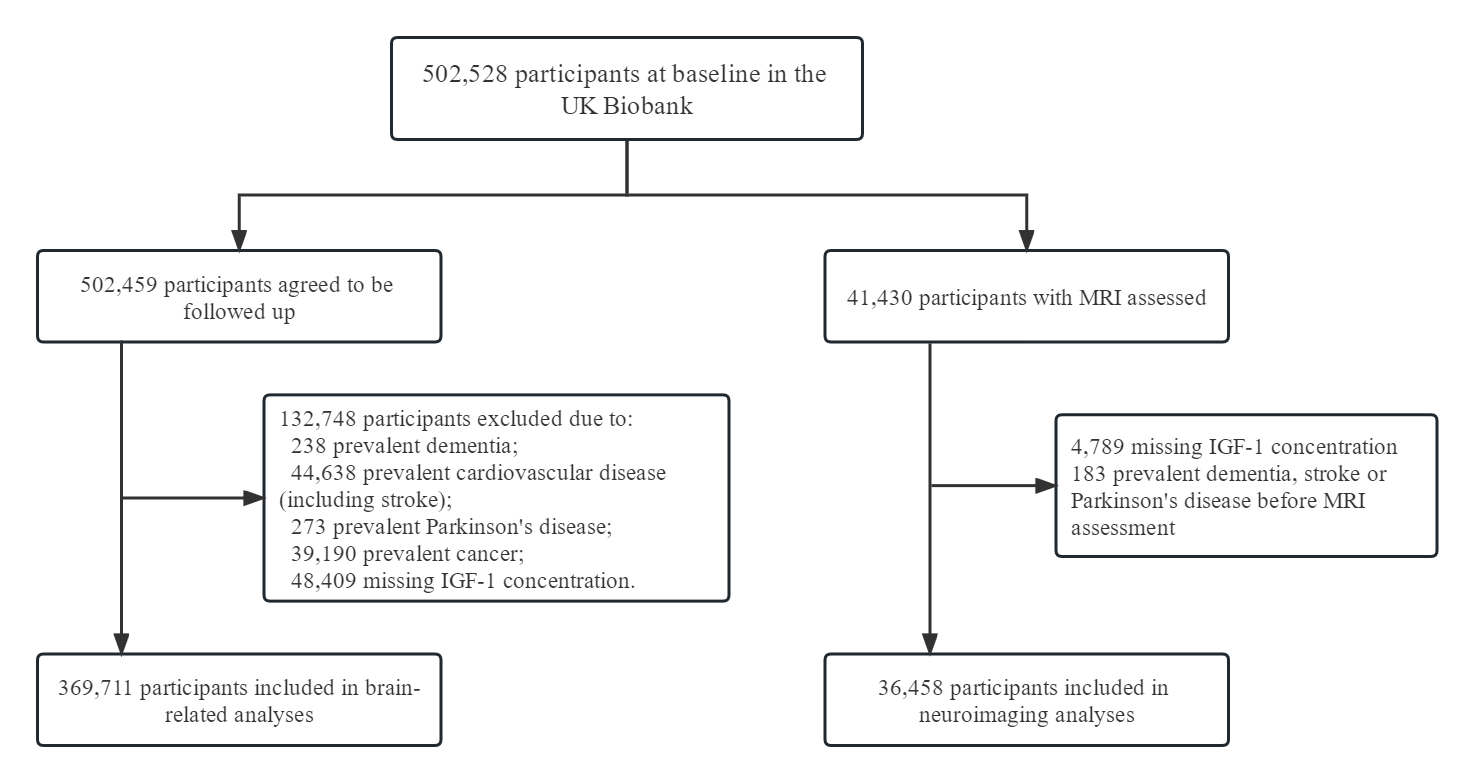
**

**Figure S1: Participants flow diagram.**


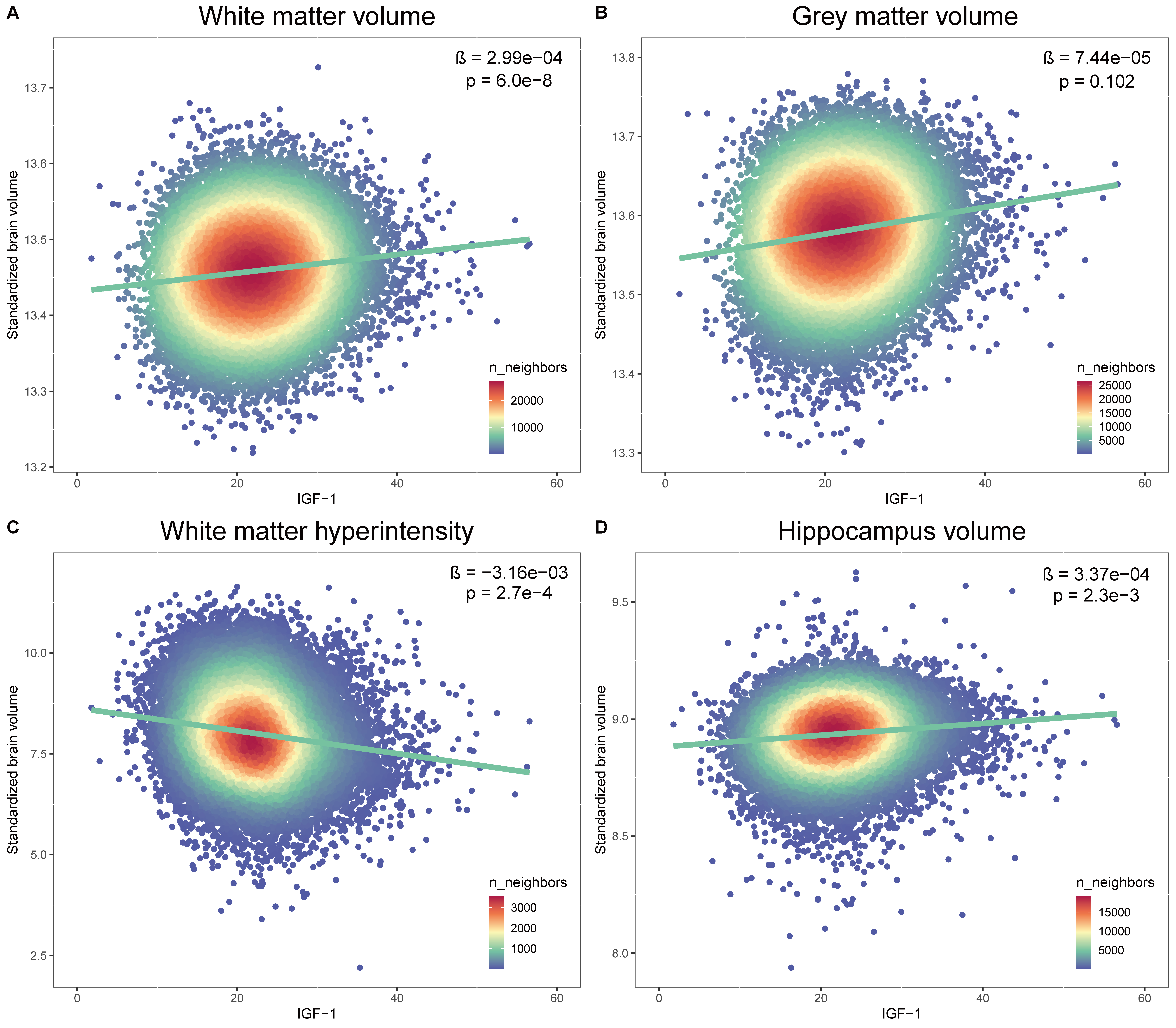
**Figure S2: Associations between IGF-1 concentrations and neuroimaging features.**

Raw data (i.e., unadjusted) are plotted. Solid lines represent estimated regression lines and shaded areas represent 95% CIs. Statistical values were obtained using multiple linear regressions controlling for sex, age, ethnicity, educational attainment, Townsend deprivation index, smoking status, alcohol intake frequency, vegetable and fruit intake, body mass index, hypertension, diabetes, total cholesterol, and C-reactive protein. Brain volume values are normalized for head size and log-transformed.


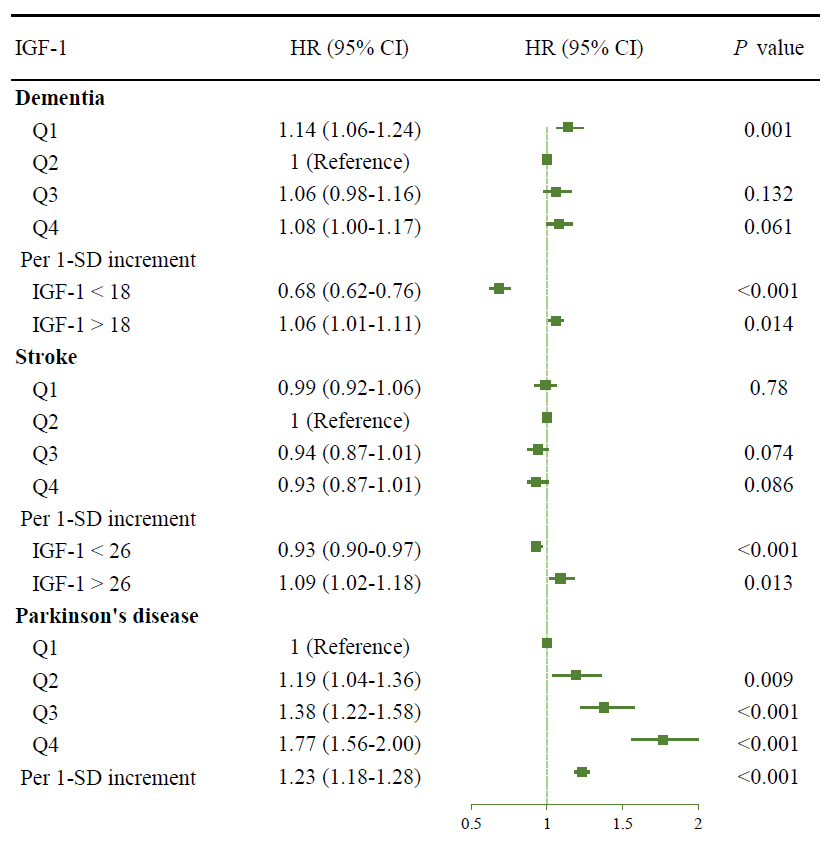
**Figure S3: The associations of IGF-1 concentrations with brain-related disorders after additional adjustment for APOE ε4 genotype.**

The analyses were adjusted for sex, age (timescale), ethnicity, educational attainment, Townsend deprivation index, smoking status, alcohol intake frequency, vegetable and fruit intake, body mass index, hypertension, diabetes, total cholesterol, and C-reactive protein.


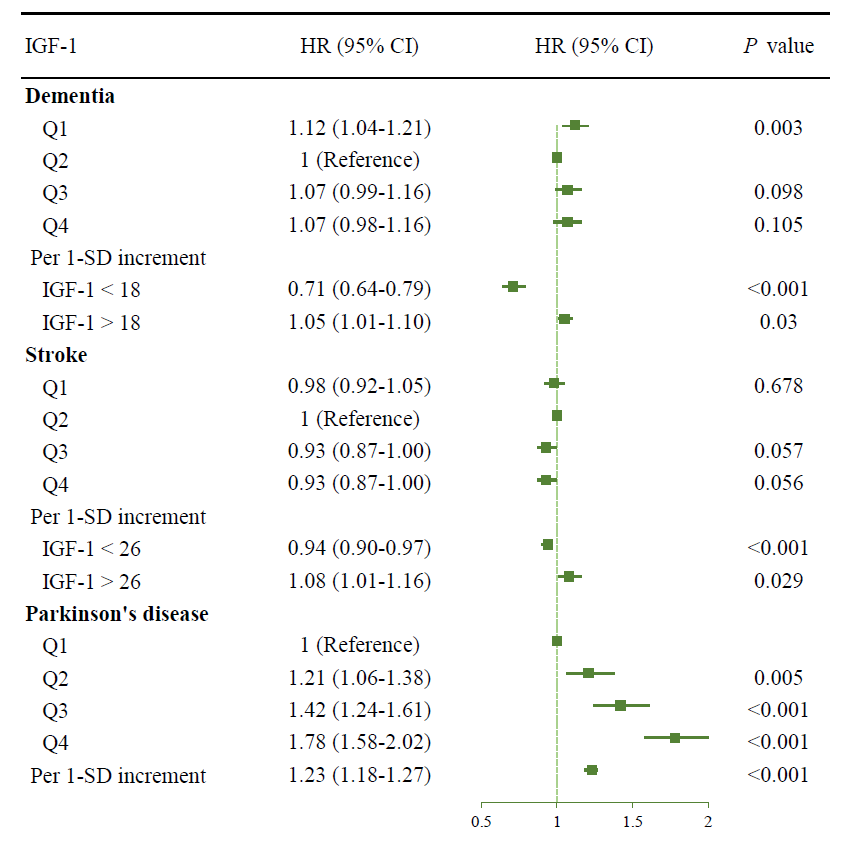
**Figure S4: The associations of IGF-1 concentrations with brain-related disorders after imputing missing covariates using multiple imputation.**

The analyses were adjusted for sex, age (timescale), ethnicity, educational attainment, Townsend deprivation index, smoking status, alcohol intake frequency, vegetable and fruit intake, body mass index, hypertension, diabetes, total cholesterol, and C-reactive protein.
